# A HELLP syndrome complicates a gestational trophoblastic neoplasia in a perimenopausal woman: a case report

**DOI:** 10.1186/s12885-016-2641-2

**Published:** 2016-08-02

**Authors:** Guillaume Vogin, François Golfier, Touria Hajri, Agnès Leroux, Béatrice Weber

**Affiliations:** 1 Department of Radiation Oncology, Institut de Cancérologie de Lorraine, Avenue de Bourgogne, 54500 Vandoeuvre Les Nancy, France; 2 Department of Obstetrics and Gynaecology, Lyon Sud University Hospital, Lyon, France; 3 French Trophoblastic Disease Reference Centre, Lyon Sud University Hospital, Lyon, France; 4 Department of Pathology, Institut de Cancérologie de Lorraine, Vandoeuvre les Nancy, France; 5 Department of Oncology, Institut de Cancérologie de Lorraine, Vandoeuvre les Nancy, France

**Keywords:** Preeclampsia, HELLP syndrome, Gestational trophoblastic neoplasia, Perimenopause

## Abstract

**Background:**

HELLP syndrome is a combination of symptoms described as hemolysis, elevated liver enzymes and low platelets, that complicates 0.01–0.6 % of pregnancies. HELLP syndrome has been scarcely reported associated with partial moles, another rare complication of pregnancy. This manuscript describes the only reported case of HELLP syndrome associated with a complete invasive hydatiform mole.

**Case presentation:**

We report a perimenopausal patient in prolonged remission from an uncommon high-risk invasive complete mole. The diagnosis was set in a context of early onset preeclampsia and HELLP syndrome. The development of life-threatening complications required primary hysterectomy. Postoperative hCG quickly returned to normal with EMA/CO multi-agent chemotherapy.

**Conclusion:**

Our patient is in prolonged remission from a complete mole complicated with EOP and HELLP syndrome. This exceptional case of complicated gestational trophoblastic neoplasia reflects a very rare condition in which several risk factors for placental ischemia are associated. Emergency hysterectomy should be considered as salvage initial treatment in such life-threatening situations.

## Background

HELLP, a syndrome characterized by hemolysis, elevated liver enzyme levels and a low platelet count, is a very rare and severe obstetric complication that usually presents in the third trimester of pregnancy [1].

In perimenopausal women, spontaneous pregnancy is rare and associated with an increased incidence of maternal complications such as pregnancy-induced hypertension and the related complications: preeclampsia and HELLP syndrome [2].

Specifically in this age group in parallel, the risk of gestational trophoblastic disease (GTD) has been reported as high as 1 in 8 pregnancies over the age of 50 - with a higher potential for malignant transformation [3–6]. GTD not only refers to premalignant entities such as complete and partial hydatiform moles (HM) but also to malignant diseases - termed gestational trophoblastic neoplasia (GTN) - such as invasive mole, choriocarcinoma, placental site and epithelioid trophoblastic tumours. Reference treatment of HM in reproductive age women is uterine evacuation while chemotherapy is indicated to treat FIGO low- or high-risk GTN [6]. In perimenopausal women, a primary hysterectomy can be recommended either as a method for uterine evacuation of HM or as a primary treatment of non-metastatic GTN with or without severe bleeding [5, 7].

## Case presentation

A 52- year old perimenopausal caucasian woman, gravida 3 para 3, with a 10-week long vaginal bleeding, bloating, fatigue, weight gain (>7 kg), and hypogastric mass was admitted to the local emergency room for an epigastric pain and a mild dyspnea. She also observed breast tenderness for the last 3 months. Her personal history included: appendectomy, amiodarone-induced hypothyroidism, chronic atrial fibrillation and breast abscess but not hypertension. Her last delivery was 23 years ago and she discontinued oral contraceptive pill at least 18 months back. She then observed hot flashes and menstrual irregularity with longer menstrual cycles and her last menses occurred 4 months ago.

**Fig. 1:**
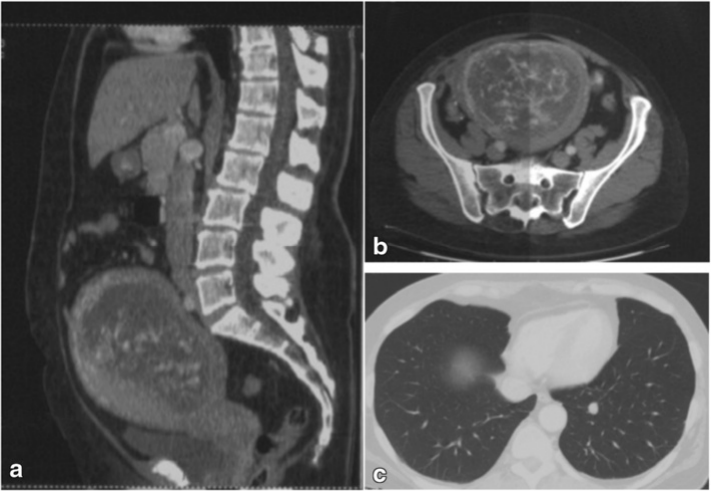
Abdominopelvic contrast-enhanced CT scans showing an enlarged uterus with focal areas of hypoattenuation; **a** mid sagittal plane; **b** transverse plan. **c** Axial chest CT scan (lung window) showing at least one left pulmonary metastase

A computed tomography (CT) scan of the chest, abdomen and pelvis showed a uterine enlargement (15 × 12 cm axial) with a heterogeneous hypodense endometrium punctuated with nodular areas enhanced by iodinated contrast (Fig. 1a & b). Neither fetus nor adnexal mass was detected. Diffuse bilateral pulmonary nodules were observed (Fig. 1c). While her serum hCG level was 0.96 × 10^6^ IU/L, an endometrial trans-cervical biopsy showed two non-malignant chorionic villi.

**Fig. 2:**
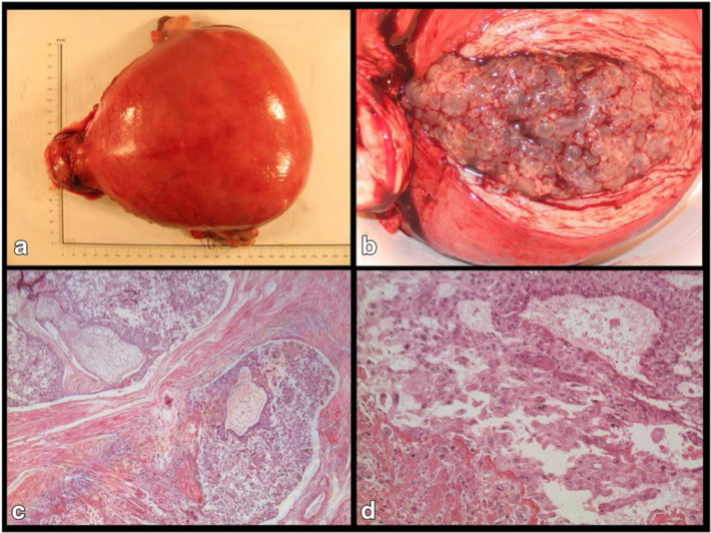
**a** Photograph of the specimen with scale (cm); **b** Photograph of the specimen with longitudinal incision exposing vesicles; **c** Low magnification micrograph of the invasive complete hydatiform mole component (hematoxylin and eosin): two enlarged chorionic villi associated with a trophoblastic proliferation invading the vessels and the muscular wall of the uterus (*bottom left*); **d** high magnification micrograph of a suspected choriocarcinomatous component (hematoxylin and eosin): large syncitiotrophoblasts associated with a proliferation of atypical intermediate cytotrophoblasts

When she was referred to the regional cancer center ten days later, she was further diagnosed with the following signs of early onset preeclampsia (EOP): new onset of severe hypertension (170/100 mmHg), proteinuria, oliguria, headache, hyper reflexivity in lower limbs and growing epigastric pain radiating to both hypochondria. While her fundal height was measured to be 18 cm, her laboratory tests including TBC, kidney and liver function were initially normal except a serum hCG test rising to 1.266 × 10^6^ IU/L. Blood pressure and diuresis were stabilized after a parenteral treatment of severe hypertension and pain. Her biological results, however, rapidly deteriorated with hemolytic anemia (hemoglobin 97 g/L, haptoglobin <0.06 g/L), thrombocytopenia (75 G/L), and elevated liver enzymes (ALT 120 IU/L, AST 252 IU/L). Additionally, she developed severe proteinuria (1.85 g/24 h). Together, she presented a complete form of HELLP syndrome. The patient was transferred to the intensive care unit and a salvage total abdominal hysterectomy with bilateral salpingo-oophorectomy was immediately decided in accordance with the French Trophoblastic Disease Reference Centre advice. Gross examination of the specimen showed an enlarged and tensed uterine body (22 × 20 × 10 cm, Fig. 2a) whose longitudinal incision released macroscopic vesicles without any identifiable fetus (Fig. 2b). Histological examination further revealed a complete and invasive hydatidiform mole (Fig. 2 c & d). Histology was reviewed within the French network of trophoblastic disease referent pathologists.

One week after hysterectomy, her biological results markedly improved (hemoglobin 105 g/L, haptoglobin 2.7 g/L, platelets 311 G/L, ALT 24 IU/L, AST 23 IU/L and hCG 31.240 × 10^3^ IU/L.

**Fig. 3:**
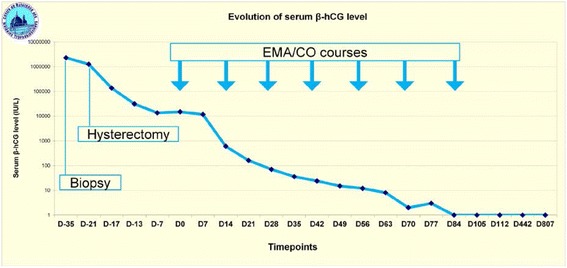
Response to treatment measured by hCG tumour marker concentration in blood

Thoraco-abdomino-pelvic CT scan, liver MRI, brain CT and 18 F-FDG PET/CT detected diffuse metastases limited to the lungs (visible on chest CT scan but not by chest-X-ray). She was thus considered to develop a post-molar high-risk GTN with a FIGO stage/score of III:7 [8]. Therefore, an EMA-CO multi-agent chemotherapy was initiated at day 20 post-operative, as the patient left the intensive care unit [9]. She was subsequently registered in the national database with her informed signed consent [10]. Complete remission of GTN was ascertained by the rapid hCG regression within 10 weeks (Fig. 3). She was administered five EMA/CO cycles with two more consolidation courses after normalization of serum hCG levels that were periodically followed up to 24 months [11]. A complete response was observed on the thoracic CT four months after diagnosis. The patient is disease-free for ten years.

## Discussion

Patients in their sixth decade are not expected to be spontaneously pregnant, and a physician may not even think of checking hCG level when confronted with abnormal vaginal bleeding. Moreover low levels of hCG production in the perimenopausal and postmenopausal state is a normal physiologic phenomenon [12]. Therefore, the diagnosis of pregnancy and, moreover, GTD may be difficult.

In the particular case of our patient, we had to face an unusual clinical presentation. While the usual presenting symptoms of perimenopausal GTD are vaginal bleeding, stigmata of pregnancy with nausea or vomiting [5, 13], our patient presented with early onset preeclampsia (EOP), a rare condition that develops during the second trimester of gestation and can complicate GTD, especially in older reproductive age women [14]. EOP is associated with a high incidence of HELLP syndrome and a 20-fold increase in maternal mortality [15, 16]. EOP is particularly difficult to diagnose when preexisting disease such as hypertension is present, especially in peri/postmenopausal women. Other less common causes of severe hypertension, including thyrotoxicosis, pheochromocytoma and recreational drug use, should be considered in the differential diagnosis. EOP and some glomerulopathies - possibly related to hypertension - may have similar clinical and laboratory findings. Conversely preeclampsia itself increases the risk of kidney disease later in life. The main differential diagnoses of HELLP include thrombotic microangiopathies (thrombotic thrombocytopaenic purpura and haemolytic uraemic syndrome (HUS) and acute fatty liver of pregnancy.

Another atypical characteristics of the disease reported here is the absence of described association between HELLP syndrome and complete hydatidiform moles, whatever the age of the patients. Only HELLP syndromes with partial moles have been scarcely reported [17–19].

## Conclusion

Our patient is in prolonged remission from a complete invasive mole complicated with EOP and HELLP syndrome. The development of such a life-threatening complication can require emergency primary hysterectomy, which can be recommended as the first curative approach of GTN when childbearing considerations have been fulfilled. Relationship of this exceptional HELLP syndrome in a GTN should be further investigated.

## Abbreviations

(β-)hCG, (β-) human chorionic gonadotropin; ALT, alanine aminotransferase; AST, alanine aminotransferase; EMA/CO, etoposide, methotrexate, and dactinomycin/cyclophosphamide and vincristine; EOP, early onset preeclampsia; GTD, gestational trophoblastic disease; GTN, gestational trophoblastic neoplasia; HELLP, Hemolysis - Elevated Liver enzymes - Low Platelet count
